# Early Retinal Flow Changes after Vitreoretinal Surgery in Idiopathic Epiretinal Membrane Using Swept Source Optical Coherence Tomography Angiography

**DOI:** 10.3390/jcm8122067

**Published:** 2019-11-24

**Authors:** Rodolfo Mastropasqua, Rossella D’Aloisio, Pasquale Viggiano, Enrico Borrelli, Carla Iafigliola, Marta Di Nicola, Agbéanda Aharrh-Gnama, Guido Di Marzio, Lisa Toto, Cesare Mariotti, Paolo Carpineto

**Affiliations:** 1 Eye Clinic, Polytechnic University of Marche, 60126 Ancona, Italy; rodolfo.mastropasqua@gmail.com (R.M.);; 2 Institute of Ophthalmology, University of Modena and Reggio Emilia, 41121 Modena, Italy; 3 Ophthalmology Clinic, Department of Medicine and Science of Ageing, University G. d’Annunzio Chieti-Pescara, 66100 Chieti, Italy; pasquale.viggiano90@gmail.com (P.V.); carlaiafigliola@hotmail.it (C.I.); gnamaomer@tiscali.it (A.A.-G.); dimarzio61@alice.it (G.D.M.); l.toto@unich.it (L.T.); p.carpineto@gmail.com (P.C.); 4 Department of Ophthalmology, University Vita Salute, IRCCS Ospedale San Raffaele, 20132 Milan, Italy; borrelli.enrico@yahoo.com; 5 Laboratory of Biostatistics, Department of Medical, Oral and Biotechnological Sciences, University G. d’Annunzio Chieti-Pescara, 66100 Chieti, Italy; mdinicola@unich.it

**Keywords:** swept source optical coherence tomography angiography, vitreoretinal surgery, idiopathic epiretinal membrane, retinal vessel density

## Abstract

(1) Background: The aim of this observational cross-sectional work was to investigate early retinal vascular changes in patients undergoing idiopathic epiretinal membrane (iERM) surgery using swept source optical coherence tomography angiography (SS-OCTA); (2) Methods: 24 eyes of 24 patients who underwent vitrectomy with internal limiting membrane (ILM) peeling were evaluated pre- and postoperatively using SS-OCTA system (PLEX Elite 9000, Carl Zeiss Meditec Inc., Dublin, CA, USA). For each eye, five 6x6-mm OCTA volume scans were acquired by two observers independently. The en face images of superficial capillary plexus (SCP) were then exported to imageJ and a semi-automated algorithm was used for subsequent quantitative analysis. Perfusion density (PD), vessel length density (VLD), vessel diameter index (VDI) and vessel tortuosity (VT) of SCP were evaluated in both the parafoveal (2.5 mm diameter) and perifoveal areas (5.5 mm diameter); (3) Results: At OCTA analysis statistically significant differences were found between controls and diseased eyes for all parameters in parafoveal and perifoveal regions (*p* < 0.001; *p* < 0.05) except for perifoveal VLD. During 6-month follow up, both anatomical/perfusion and functional parameters showed a statistically significant improvement if compared to preoperative values. In detail, at one-month post vitrectomy, VLD and VT significantly changed in parafoveal region (*p* = 0.043; *p* = 0.045), while PD and VDI showed a trend of increase in both parafoveal and perifoveal region. At 6 months after surgery, PD, VLD and VT of parafoveal region significantly improved (*p* = 0.021, *p* = 0.018, *p* = 0.047 respectively). (4) Conclusions: SS-OCTA provides a quantitative and qualitative analysis of the superficial capillary plexus allowing for early vascular changes assessment after vitrectomy with iERM and ILM peeling.

## 1. Introduction

Epiretinal membrane (ERM) is a common macular disease characterized by proliferation of abnormal tissues on the surface of the macula, at the interface between the vitreous and the retina [1,2,3]. Based on previous studies, ERMs can be associated with other ocular disorders, typically diabetic retinopathy and retinal vein occlusion, or can develop after cataract surgery or vitrectomy for retinal detachment [4,5,6]. On the other hand, ERMs are defined as idiopathic ERM (iERM) when no cause can be found [7]. Importantly, iERM aetiology is still unclear, although seems to be related to an anomalous posterior vitreous detachment (PVD) [8,9]. Some pathophysiological theories of iERM formation support a glial tissue origin. It has been suggested that after the onset of PVD, retinal glial cells deriving from Müller cells or astrocytes, proliferate and migrate, resulting in the fibrocellular tissue formation characterizing the scaffold of ERM recently formed [7,9]. Fibroblasts and the myofibroblasts, which are supposed to derive from Müller’s cells, hyalocytes or retinal pigment epithelium (EPR), are the most represented cells of the most advanced ERM stages characterized by a rich tractional component.

Idiopathic ERM is still a frequent cause of visual impairment in working age population, due to traction of the membrane leading to the formation of retinal folds and consequently retinal thickening [1,7].

To date, optical coherence tomography angiography (OCTA) has been introduced in ERM evaluation, allowing a detailed assessment of retinal vascular plexuses and highlighting structural and functional changes related to ERM presence preoperatively and modifications of retinal flow after its removal [10,11,12,13,14]. 

The introduction of high-speed swept source (SS) OCT devices provided more details about retinal diseases, highlighting the reorganization of the inner retinal layers visible in eyes with advanced stage ERM [11,15,16,17]. 

The recent development of SS-OCTA, thanks to use of a longer wavelength and a higher speed, allows a wider retinal field of view of superficial capillary plexus (SCP), deep capillary plexus (DCP) and choriocapillaris (CC) and a better visualization of deep layers such as CC and choroid [17,18]. 

The goal of this study was to report early SCP vascular changes following idiopathic epiretinal membrane surgery using a SS-OCTA device. Notably, these changes were determined in two different macular regions comparing the preoperative and post-operative vascular modifications. 

## 2. Experimental Section

### 2.1. Study Participants

In this observational cross-sectional study, 24 subjects with a unilateral idiopathic ERM were enrolled at the Ophthalmology Clinic of University G. d’Annunzio, Chieti-Pescara, Italy. Twenty-four eyes of healthy people were considered as controls. The study was approved by our Institutional Review Board (IRB) (Department of Medicine and Science of Ageing, University G. d’Annunzio Chieti-Pescara) and adhered to the tenets of the Declaration of Helsinki. An IRB approved informed consent was obtained from all patients. All subjects with iERM underwent 25 G pars plana vitrectomy with ERM and internal limiting membrane (ILM) peeling and were imaged with the PLEX Elite 9000 device (Carl Zeiss Meditec Inc., Dublin, CA, USA) between January 2018 and January 2019. Moreover, all patients received a complete ophthalmologic examination, which included the measurement of best corrected visual acuity (BCVA), intraocular pressure (IOP) and ophthalmological evaluation. Inclusion criteria were: (i) diagnosis of iERM (stage 3, according to Govetto classification system) [19], (ii) no history of previous ocular surgery, except for cataract surgery, (iii) iERM duration ≤ 6 years. Exclusion criteria were: (i) evidence or history of ocular conditions such as retinal detachment, retinal vascular occlusions, uveitis, high myopia, trauma; (ii) evidence or history of systemic disorders, including diabetes and systemic hypertension; (iii) poor image quality.

### 2.2. Image Acquisition

Subjects underwent OCTA imaging using the PLEX Elite 9000 device (Carl Zeiss Meditec Inc., Dublin, CA, USA) which uses a swept laser source with a central wavelength of 1050 nm (1000–1100 nm full bandwidth) and operates at 100,000 A-scans per second. For each eye, five 6x6 -mm OCTA volume scans were acquired by two independent graders (RM and RDA), preoperatively and at 1 month after surgery. FastTrac motion correction software was used while the images were acquired. 

Poor quality images (signal strength index (SSI) < 8) with either significant motion artifact or incorrect segmentation were excluded. All selected images were carefully visualized by the two retinal specialists independently to ascertain the correctness of segmentation and in case of erroneous recognition by the software of the position of the boundaries of the ILM and retinal pigment epithelium (RPE) manual correction was performed using the segmentation and propagation editing tool from the device. 

### 2.3. Image Processing

**Figure 1:**
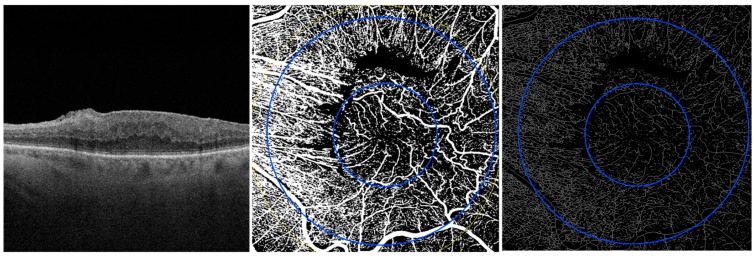
OCT scan and en face OCTA images of SCP (on the left), binarized SCP (in the middle) and skeleton SCP (on the right) before vitrectomy of 3-stage ERM.

**Figure 2:**
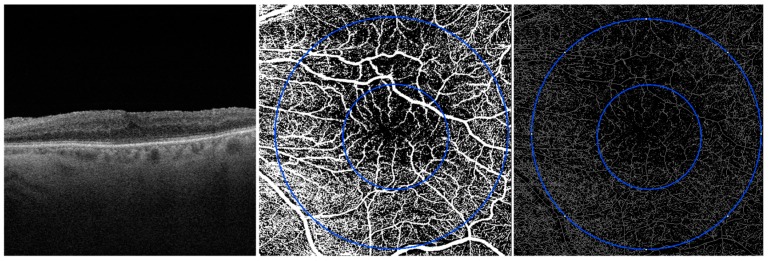
OCT scan and en face OCTA images of SCP (on the left), binarized SCP (in the middle) and skeleton SCP (on the right) 1 month after vitrectomy.

**Figure 3:**
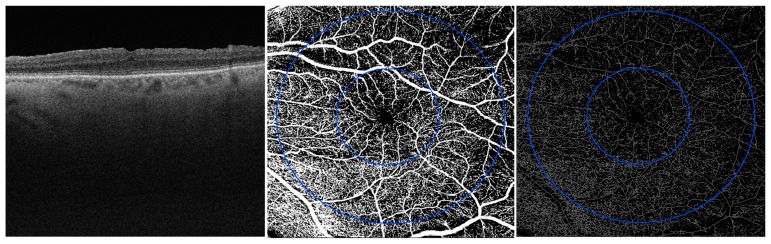
OCT scan and en face OCTA images of SCP (on the left), binarized SCP (in the middle) and skeleton SCP (on the right) 6 months after vitrectomy.

The main outcome measures were: (i) SCP perfusion density (PD); (ii) SCP vessel length density (VLD); (iii) SCP vessel diameter index (VDI); (iv) SCP vessel tortuosity (VT). In order to quantify these variables, a slightly modified previously reported semi-automated algorithm was employed [20,21]. In brief, for each eye, en face OCTA images segmented at the SCP level were imported into ImageJ software version 1.50 (National Institutes of Health, Bethesda, MD; available at http://rsb.info.nih.gov/ij/index.html) and, consequently were processed with a ‘‘top-hat’’ filter. Each image was duplicated and two different binarizarion methods were then performed on the 2 resultant images: (i) 1 image was first processed by a ‘‘hessian’’ filter, followed by global thresholding using the ‘‘Huang’s fuzzy’’ method; (ii) the other (duplicate) image was binarized using the ‘‘median local’’ thresholding. Finally, the two obtained images were combined. Perfusion density was thus calculated as a unitless proportion of the number of pixels over the threshold divided by the total number of pixels in the analyzed area. Successively, the SCP images obtained after binarization were skeletonized and these images were employed to measure VLD [21,22]. In order to measure the average vessel caliber, we calculated VDI by dividing the area in the binarized image by that in the skeletonized image. Finally, using the “Analyze skeleton” plugin, the actual length of each branch and the imaginary straight length between two branch nodes—points of connections—were marked. We calculated VT by dividing the sum of actual branch lengths by the sum of straight lengths between branch nodes [23]. The quantitative analysis was thus performed in the macular region, which was defined as a circular annulus around the fovea with diameter of 5.5 mm and excluding the foveal avascular zone (FAZ). Furthermore, the analysis of the macular region was further divided into the parafoveal and perifoveal areas (with diameters of 2.5 mm, and 5.5 mm, respectively) and was performed at the baseline (Figure 1) and after surgery (Figure 2 and Figure 3). 

### 2.4. Surgical Procedure

All diseased eyes enrolled underwent 25G 3-port pars plana vitrectomy with ERM and ILM peeling after ERM and ILM staining with a combination of 0.15% trypan blue, 0.025% brilliant blue G, and 4.00% polyethylene glycol (MembraneBlue-DualTM, DORC International, Zuidland, the Netherlands). All procedures were performed by a single and experienced surgeon (R.M.). Four eyes were phakic and twenty of them were pseudophakic. In phakic eyes combined phacovitrectomy with intraocular lens implantation in the capsular bag was performed. No intra and postoperative complications were reported.

### 2.5. Statistical Analysis

The quantitative variables were summarized as mean and standard deviation (SD) according to their distribution and qualitative variables as frequency and percentage. A Shapiro-Wilk’s test was performed to evaluate the departures from normal distribution for each variable. 

Differences in baseline demographic and clinical characteristics between control group and iERM patients group were tested by Mann-Whitney U test and Pearson chi-square test for continuous and categorical variables, respectively. Only in iERM patients group, Friedman test was applied for assessing significance differences in the quantitative variables between baseline values and follow-up measurements. Wilcoxon U test with Bonferroni correction was applied to evaluate a multiple comparison between different time points.

Lin’s concordance correlation coefficient (CCC) with the 95% confidence intervals was calculated to assess the interobserver reproducibility of image acquisition.

Spearman’s Rho correlation coefficient was applied to evaluate the linear correlation among CMT and retinal perfusion variables in iERM patients group. 

All statistical analyses were performed using R Statistical Software (version 3.5.3; R Foundation for Statistical Computing, Vienna, Austria). In all statistical tests the threshold of statistical significance was assumed equal to *p* = 0.05.

## 3. Results

### 3.1. Characteristics of Diseased and Healthy Eyes at the Baseline

A total of 24 eyes of 24 people (16 females, 8 males; mean age of 58.9 ± 8.6 years) with a diagnosis of 3-stage iERM were considered in the analysis. Clinical and demographic characteristics of diseased eyes enrolled are reported in Table 1. 

A group of 24 healthy eyes of 24 subject were considered as controls (14 females, 10 males; mean age of 55.9 ± 8.7 years). No statistically significant difference was found between diseased and normal eyes in terms of age, gender and axial length (*p* > 0.05; Table 1). 

The average central macula thickness (CMT) and average logMAR BCVA of iERM group were 512.3 ± 23.4 µm at baseline and 0.78 ± 0.38 LogMAR at baseline respectively (Table 2). 

At OCTA analysis statistically significant differences were found between controls and diseased eyes for all perfusion parameters in both parafoveal and perifoveal regions except for perifoveal VLD (Table 3).

### 3.2. Comparison between Pre-Operative and Post-Operative Morphological and Functional Parameters of Diseased Eyes

At 6 months after surgery, both anatomical/perfusion and functional parameters showed a statistically significant difference compared to the baseline values (Table 2; Figure 3).

In detail, the logMAR BCVA of iERM group improved to 0.53 ± 0.34 LogMAR and to 0.32 ± 0.40 LogMAR at 1 month and 6 months after surgical treatment respectively (Table 2). 

During the 6-month follow-up, CMT significantly decreased in iERM eyes in comparison with preoperative values (at 1-month follow up: 374.5 ± 31.7 µm, *p* < 0.001; at 6 month follow up: 374.0 ± 49.3 µm; *p* < 0.001; Table 2).

**Figure 4:**
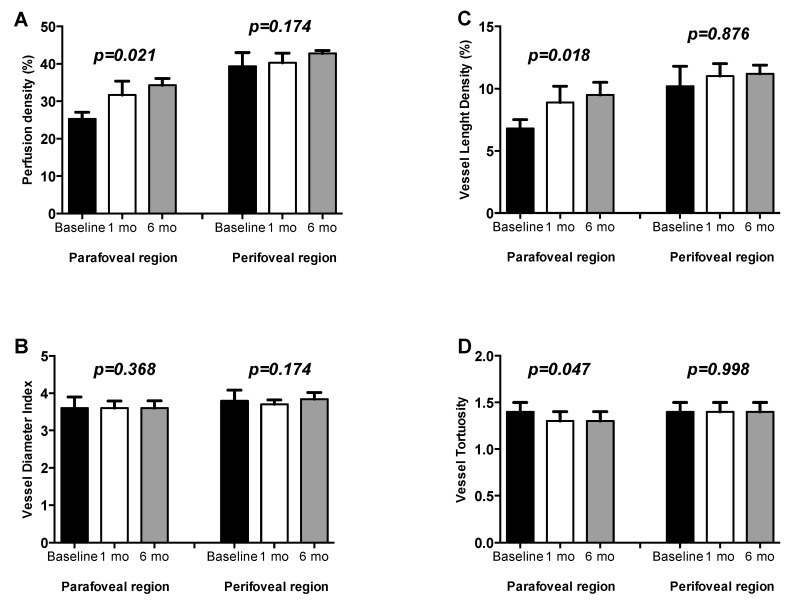
Mean and standard deviation of retinal perfusion variables in iERM patients group at baseline, at 1-month (1mo) and 6-months (6mo) follow-up after vitreoretinal surgery: (**A**) Perfusion Density (%); (**B**) Vessel Diameter Index; (**C**) Vessel Length Density (%); (**D**) Vessel Tortuosity. *p*-values reported in figure are relative to comparison between values evaluated by Friedman test.

At OCTA analysis outcome measures of both groups were compared at baseline, 1 month and 6 months after surgery. In detail, at one-month post vitrectomy, VLD and VT significantly changed in parafoveal region (*p* = 0.043; *p* = 0.045), while PD and VDI showed a trend of increase in both parafoveal and perifoveal region (Figure 4). At 6 months after surgery PD, VLD and VT of parafoveal region significantly improved (*p* = 0.021, *p* = 0.018, *p* = 0.047 respectively, Figure 4).

On the contrary, no statistically significant difference was observed in terms of PD, VLD and VT in perifoveal region before and after surgery for whole 6-month follow up (*p* = 0.174, *p* = 0.876, *p* = 0.998 respectively, Figure 4). 

Finally, in the iERM group, no significant correlations were found between anatomical and functional parameters, except for the vessel tortuosity that was significantly positively associated with CMT at 6-months (Rho = 0.674 and *p* = 0.018). The agreement between the two observers was excellent with a Lin’s CCC of 0.99 (95% CI: 0.98–0.99) in all analyzed parameters.

## 4. Discussion

It has been hypothesised that Müller cells play a main role in the pathological mechanism of ERM formation and in the support of foveolar structure. Their strong adhesion with ILM has been associated with a higher risk of ultrastructural damages of deep portions of the retina after peeling [24].

It has been widely reported a macular contraction and retinal vessel displacement in patients with a diagnosis of ERM due to tangential and centripetal forces exerted from ERM itself [18].

Tangential and vertical macular tractions are considered responsible of foveal microarchitectural changes in the retinal vasculature with vessel occlusion and tortuosity [24]. 

The tangential force would drag the superficial retinal layers away from their original location thus straightening or curling retinal vessel of superficial capillary plexus [25].

ERM removal should release all tractions, thus letting main retinal vessels and capillaries come back to their original position. However, some forces of traction could persist also after vitrectomy [26].

In addition, ILM peeling procedure has been considered to be responsible of increased elasticity of the retina thus causing a foveal displacement of capillaries [26].

Kumagai et al [27] described a centripetal movement of the inner retinal layer after ILM peeling with a centripetal shift of foveal capillaries. Furthermore, authors reported changes in ganglion cell complex, where SCP is located, with its thickness reduction, due to a partial structural restoration after ILM peeling surgery and a likely disruption of the ganglion cell neurites included in the iERM [28].

The non-invasive nature of OCTA technique has progressively and quickly gained much interest in clinical practice. OCTA is rapidly becoming a new important imaging modality for the retina/choroid, optic nerve head and even the anterior segment. It has shown a high reliability in perfusion assessment of the superficial retinal vasculature [29,30].

As already known, retinal vasculature is composed by three plexuses: the superficial, the intermediate and the deep capillary plexus. In detail, the superficial capillary plexus, located in the ganglion cell layer and nerve fiber layer, is characterized by a centripetal pattern vessel; while the vessels of deep capillary plexus, that is located in the inner nuclear layers, have typically a concentric distribution with vertical interconnections [31].

The recently introduced widefield SS-OCTA is able to provide quantitative and qualitative information of microvasculature of all three retinal capillary plexuses in central area and in midperiphery.

SS-OCTA uses tunable laser centered at 1060 nm and with at a scan speed of 100,000 A scans per second and an axial resolution of 6.3 μm. This device is based on optical micro angiography complex algorithm to analyse retinal microvasculature in detail and in depth.

We aimed at evaluating retinal microvasculature parameters of superficial capillary plexus of a 6x6 mm scan area to identify early vascular changes at baseline and after vitrectomy with ILM peeling in terms of parafoveal and perifoveal retinal perfusion using SS-OCTA.

In detail, at SCP level, PD, VDI, VLD and TV were investigated. We decided to focus only on the superficial plexus in order to avoid ERM related projection artefacts. Indeed, traction of ERM and macular edema may alter OCTA signal quality of deep capillary plexus status. Although artefacts can be corrected with projection removal algorithms, OCTA remains susceptible to projection artefacts because of the superficial blood flow, thus leading a difficult interpretation of deep retinal vasculature feature with a potential loss of details [32].

Furthermore, ERM with rich intraretinal fluid component weakens the reflected OCTA signal intensity from deeper layers, although swept-laser source allows more light to penetrate deeper tissue due to the reduced scattering properties of tissue [32].

In our cohort we included only ERM of stage 3 to eliminate possible bias related to different severity of the disease. In addition, we considered patients with a similar duration of ERM diagnosis that was a mean of ≤ 5.2 ± 0.7 years.

A significant reduction in capillary blood flow velocity of iERM patients was observed using fluorescein angiography if compared with healthy age-matched eyes probably due to increase of venous resistance caused by vessel abnormalities [25,33].

Six months after vitrectomy, a statistically significant improvement in blood flow velocity was reported by the authors, related to capillary vessel recanalization [25,33].

In our cohort, PD and VLD in iERM group were significantly different if compared to the control group likely due to a centripetal movement of microvessels exerted from traction with central vessel crowding. It can be hypothesized that partial capillary sub- /occlusion occurred related to ERM presence thus causing flow impairment in the foveal region [25]. Conversely, Nelis and coworkers [34] found a significant increase of the macular vessel density ratio (vessel density of the foveal and parafoveal region) of MER eyes in comparison to healthy controls, that presumably reflected a vascular displacement from the perifoveal to the foveal area without a significant vessel occlusion component. 

The degree of perifoveal hemodynamic changes is strictly associated with severity of ERM and depth and duration of traction, for this reason we preferred to evaluate eyes with the same ERM grade and with a duration of ≤ 6 years from the first diagnosis.

At 1-month post-surgery, although a trend of increase was observed, overall macular PD of iERM group did not show any significant difference compared to preoperative values, both in perifoveal and parafoveal areas, probably because of the recanalization of occlused microvessels contemporaneously with the decrease of central vessel crowding toward perifovea. 

On the contrary, parafoveal VLD significantly increased at 1-month follow-up. The vessel length density increase was likely due to a reopening of little vessels that were sub-occlused in the preoperative period because of ERM traction release. 

We speculate that forces exerted from ERMs could have a greater impact on tiny little vessels leading to a temporary sub-/occlusion of them. After vitrectomy these capillaries would open again due to the release of tractions as well as a recovery to the vessel original positions. Dell’Omo et al [35] have previously described as, after vitreoretinal surgery for retinal detachment, vessels seen with autofluorescence did not come back to their original position, on the contrary they would assume a new position. 

Our results showed an initial increase in vessel diameter in both parafoveal and perifoveal regions because of the reopening and recanalization of microvessels, thus OCTA signal would consider also diameter of reopened smaller vessels in the overall analysis.

At 6-month follow up after vitrectomy, both anatomical/perfusion and functional parameters showed a statistically significant improvement if compared to the baseline values, confirming that the real recanalization of parafoveal microvasculature needs a longer time to completely recovery.

Finally, our work investigated vascular tortuosity and it appears to be the first study describing this parameter in patients with iERMs using SS-OCTA.

A relatively recent study [35] has identified VT detected with OCTA as a useful quantitative measure correlated to diabetic retinopathy (DR) stage. It has been reported that a higher VT is associated with a higher progression to proliferative diabetic retinopathy. Therefore, VT seems to be an early indicator of microvascular damages in the retina and a quantitative marker to monitor the progression of DR [36].

In our work VT showed a statistically significant decrease postoperatively compared to preoperative values only in the parafoveal area. The latter region is probably mainly involved in the early modifications of VT recovery after surgery. Perifoveal area would need more time to a complete anatomical recovery. Although surgical treatment partially resolves tangential and vertical retinal tractions, thus improving macular microcirculation thanks to recanalization of capillary vessels, some tractional effects may persist also after surgery, explaining no significant change of perifoveal VT. 

To the best of our knowledge this is the first study that analyses in detail retinal microvasculature features, quantifying contemporary perfusion and tortuosity as an elaboration of a single scan acquisition. 

The comprehension of early changes of retinal circulation could be predictive of macular status at baseline and of macular morphology and function after surgery.

With further advancement in imaging technology, OCTA may serve as an alternative non-invasive device to the traditional fluorescein angiography in order to better identify ERM features and to predict visual prognosis after surgery.

In terms of functional changes, our findings showed a significant early improvement in terms of visual outcome and CMT postoperatively, already starting from one month after surgery.

Our results suggest a relationship between early changes of capillary architecture after vitrectomy and postoperative retinal function in iERM patients. This issue could be helpful to better identify the pathophysiology of the disease, and to assess morphological markers (vascular) that could be helpful to predict the postoperative outcome. 

The main limitation of this study is the relatively small sample and the cross sectional nature of analysis. The follow up was very small, nevertheless we wanted to focus intentionally on early changes after surgery, to identify predictive flow modifications of the macula over time. A longer follow up of the same parameters assessed with the same new swept source device is needed to possibly predict a prognosis of the final anatomical and functional success, by analyzing and following depth-selectively vessel behavior pre and postoperatively.

In conclusion, our cohort of patients with iERM showed very early (1 month post-surgery) macular hemodynamic changes such as vessel tortuosity and vessel length density with a micro architectural restoration of vessels. On the other hand, macular perfusion density would need a longer time (6-month follow-up) to significantly improve, likely due to a gradual and progressive reopening and recanalization of microvessels.

## Figures and Tables

**Table 1:** The clinical and demographics characteristics of diseased eyes (iERM group) and healthy eyes (control group).

Variable	iERM Group	Control Group	*p*-Value
Age (years), mean ± SD	58.9 ± 8.6	55.9 ± 8.7	0.326 ^a^
Gender (male/female), *n* (%)	8 (33.3)/16 (66.7)	10 (41.7)/14 (58.3)	0.766 ^b^
Axial length (mm), mean ± SD	22.9 ± 1.1	23.1 ± 0.5	0.422 ^a^
ERM 3 stage, *n* (%)	24 (100.0)	-	*-*
Duration of ERM(years), mean ± SD	5.2 ± 0.7	-	*-*
Phakic/pseudophakic, *n (%)*	4 (17.0)/20 (83.0)	24 (100.0)	*-*

^a^ Mann-Whitney U test; ^b^ Pearson chi-square test.

**Table 2:** Anatomical and functional parameters of healthy (control group) and diseased eyes (iERM group).

Variable	iERM Group				Control Group	
	Baseline	1 Month	6 Months	Friedman *p*-Value	Baseline	Mann–Whitney *p*-Value
**CMT (µm)**	512.3 ± 23.4	374.5 ± 31.7 *	374.0 ± 49.3	<0.001	185.9 ± 12.9	<0.001
**logMAR BCVA**	0.78 ± 0.38	0.53 ± 0.34	*0.32* ± *0.40*	<0.001	0.10 ± 0.41	<0.001

* *p* < 0.05 vs. previous time point.

**Table 3:** SCP analysis of parafoveal and perifoveal region at baseline of iERM and healthy groups.

Variable	Parafoveal Region			Perifoveal Region		
	iERM Group	Control Group	Mann-Whitney *p*-Value	iERM Group	Control Group	Mann-Whitney *p*-Value
**Perfusion Density (%)**	25.3 ± 1.8	30.6 ± 4.4	<0.001	39.4 ± 3.6	42.3 ± 1.8	0.023
**Vessel Length Density (%)**	6.8 ± 0.7	8.8 ± 1.3	<0.001	10.2 ± 1.6	11.3 ± 0.7	0.105
**Vessel Diameter Index**	18.5 ± 1.9	21.7 ± 3.2	0.009	29.1 ± 2.3	31.0 ± 1.3	0.023
**Vessel Tortuosity**	1.4 ± 0.1	0.7 ± 0.1	<0.001	1.4 ± 0.1	0.7 ± 0.1	<0.001
